# Determination of picomolar levels of methylmercury complexes with low molecular mass thiols by liquid chromatography tandem mass spectrometry and online preconcentration

**DOI:** 10.1007/s00216-020-02389-y

**Published:** 2020-01-16

**Authors:** Van Liem-Nguyen, Hoang-Tung Nguyen-Ngoc, Gbotemi A. Adediran, Erik Björn

**Affiliations:** 1 Laboratory of Advanced Materials Chemistry, Advanced Institute of Materials Science, Ton Duc Thang University, 19 Nguyen Huu Tho Street, Tan Phong Ward, District 7, Ho Chi Minh City, Vietnam; 2 Department of Chemistry, Umeå University, 901 87 Umeå, Sweden

**Keywords:** Methylmercury-thiol complex, Low molecular mass thiols, Liquid chromatography tandem mass spectrometry, Online preconcentration

## Abstract

Methylmercury (MeHg) is one of the most potent neurotoxins. It is produced in nature through the methylation of inorganic divalent mercury (Hg^II^) by phylogenetically diverse anaerobic microbes. The mechanistic understanding of the processes that govern the extent of bacterial export of MeHg, its bioaccumulation, and bio-toxicity depends on accurate quantification of its species, especially its complexation with low molecular mass thiols; organometallic complexes that are difficult to detect and measure in natural conditions. Here, we report the development of a novel analytical method based on liquid chromatography tandem mass spectrometry (LC-MS/MS) to determine 13 MeHg complexes with important thiol compounds which have been observed in the environment and in biological systems. By using online preconcentration via solid phase extraction (SPE), the method offers picomolar (12–530 pM) detection limits, the lowest reported so far for the determination of MeHg compounds. Among three different SPE materials, a weak cation exchange phase showed the best efficiency at a low pH of 2.5. We further report the presence of MeHg-cysteine, MeHg-cysteamine, MeHg-penicillamine, MeHg-cysteinylglycine, and MeHg-glutamylcysteine as the predominant MeHg–thiol complexes in the extracellular milieu of an important Hg^II^ methylating bacterium, *Geobacter sulfurreducens PCA*, exposed to 100 nM of Hg^II^.

**Electronic supplementary material:**

The online version of this article (10.1007/s00216-020-02389-y) contains supplementary material, which is available to authorized users.

## Introduction

Methylmercury (MeHg) is ingested by humans mainly via rice and fish consumption [1, 2] and can damage the central nervous system and kidney and is especially dangerous for fetuses and infants [3]. The MeHg ion (CH_3_Hg^+^) has a strong affinity for soft base ligands, especially inorganic and organic reduced sulfur compounds (sulfide and thiols, respectively) [4], and the chemical speciation of MeHg in environmental and biological systems is largely dominated by complexes with sulfide, and low or high molecular mass thiols [4–6]. Low molecular mass (LMM) thiols are important in biological processes due to the complexation ability of the thiol functional group with metal ions and subsequent bio-transformations between the thiols and disulfides through redox reactions mediated by enzymatic activities [7, 8]. LMM thiols are directly synthesized by living organisms and/or indirectly formed through the reaction of sulfide with unsaturated natural organic matter in the environment [9–11]. Such thiol compounds are typically found at nM to μM concentrations in the environments, but they have also been found in mM concentrations [12–14]. Even when present at low concentrations, LMM thiols play important roles in MeHg speciation [5, 13].

Intracellular methylation of inorganic divalent mercury (Hg^II^) by widely spread anaerobic microbes carrying the *hgcA* and *hgcB* genes is generally being accepted as the main source of MeHg formation and release into the environment [15–19]. Significant increases in the Hg^II^ methylation rate have been reported for bacteria culture assays amended with specific LMM thiols [20, 21] and subsequent cellular export of MeHg has been observed to be facilitated by addition of cysteine [22]. In a recent study, a characterization of the extracellular milieu of *Geobacter sulfurreducens PCA*, an iron reducing bacterium capable of Hg^II^ methylation, revealed metabolically active biosynthesis of various LMM thiol compounds [11]. Despite the scientific evidences of the important roles of LMM thiols for MeHg cycling, the measurement of specific MeHg–thiol complexes has been hampered by constraints in analytical measurement techniques, and the behavior of specific MeHg–thiol in the environment as well as in biological systems remains poorly investigated.

Methylmercury and particularly Hg^2+^ have a strong affinity for reduced sulfur group in thiol compounds. The typical ranges of stability constants (log K) for the formation of MeHg–thiol and Hg(thiol)_2_ complexes are 16.0–17.5 and 34.6–42.1, respectively [23, 24]. Fast reaction kinetics between MeHg or Hg^2+^ and thiols have been reported using nuclear magnetic resonance spectroscopy [24–26]. Synchrotron X-ray fluorescence mapping has been combined with Hg L_III_ X-ray absorption near-edge spectroscopy to constrain the localization and speciation of Hg and MeHg in rice grain. It was shown that MeHg is present in rice bran predominantly as MeHg bound to cysteine. The MeHg–cysteine complex behaves like a mobile nutrient and is actively transported to the endosperm of rice grains during seed ripening [27]. The binding of MeHg to cysteine has also been demonstrated in fish muscle [28]. Furthermore, addition of thiols e.g., cysteine, has been shown to either enhance or suppress MeHg formation in bacteria culture assays depending on the added concentration [20, 29].

Previous attempts to elucidate reactions and processes of MeHg–thiols have mainly been based on thermodynamic modeling of the concentrations of such complexes [5]. Relying only upon thermodynamic modeling is however limited by uncertainties in stability constants and by potential kinetic constraints [24, 30]. Most analytical methods in mercury biogeochemistry research have been developed for the determination of the concentration of total mercury (Hg), elemental Hg (Hg^0^), or total MeHg (i.e., sum concentration of all MeHg species), with very few being dedicated to direct measurement of individual MeHg species [31–33]. Gas chromatography (GC) has been widely used as separation technique due to its advantages of high peak resolution and short analysis time. However, ionic metal complexes must typically be derivatized prior to separation in which case information of metal complex speciation is lost. Liquid chromatography (LC) is an alternative technique with the possibility of maintaining metal-ligand information [31]. Krupp et al. [34] reported a method for determination of MeHg and Hg^II^ complexes with cysteine (Cys) and glutathione (GSH) by LC electrospray ionization mass spectrometry (ESI-MS) and LC combined with inductively coupled plasma mass spectrometry (ICPMS). The method focused on complex identification whereas limits of detection (LODs) were not reported and losses of complexes during the determination was observed but not resolved. Bouchet and Björn [35] reported an analytical method to directly measure specific MeHg–thiol complexes based on LC-ICPMS. Although the method was successful in measuring some MeHg–thiol complexes with relatively low LODs ranging from 0.6 to 7.4 nM, it suffered from limited selectivity due to retention overlaps particularly for highly hydrophilic complexes [35]. The sensitivity of LC-ICPMS methods is generally high with respect to the Hg signal but different MeHg complexes are identified by chromatographic retention time only. This causes a relatively large risk for peak overlaps and misidentification in samples with many thiol compounds, in particular if the sample also contains a substantial concentration of Hg^II^ forming Hg^II^-thiol complexes [12, 35]. In bacteria culture and environmental systems, many different LMM thiol compound are often present [5, 11] and the development of a highly selective and more robust method is therefore essential to characterize MeHg–thiol complexes at concentrations relevant for culture systems with Hg^II^ methylating bacteria and for the environment.

In this study, we present a novel methodology for the determination of a comparatively large number of MeHg complexes (13 complexes) with LMM thiol ligands which has been frequently found in bacteria culture assays and in terrestrial and aquatic ecosystems [5, 11, 12, 21, 35]. The method is based on LC coupled with ESI triple quadrupole tandem mass spectrometry (ESI-MS/MS) with selective reaction monitoring (SRM) detection mode. The identification of the complexes relies on (i) the specific retention time on the LC column and (ii) parental mass and product ions with optimized fragmentation condition for each complex. Combined with online preconcentration by solid phase extraction (SPE), the method offers LODs at the pM level for all investigated complexes, which is the lowest LODs reported up to date for MeHg complexes. The effect of different SPE materials, pH, and sample matrix composition on the recovery of the complexes were investigated and optimized. The stability over time of the complexes was also investigated at different storage temperature. The optimized method was then applied to characterize MeHg–thiol complexes in the extracellular milieu of metabolically active *G. sulfurreducens PCA*, one of the most studied sub-surface Hg^II^ methylating bacteria in nature, amended with 100 nM HgCl_2_.

## Experimental

### Chemicals and reagents

Methylmercury chloride (MeHgCl) was purchased from Sigma-Aldrich (PESTANAL analytical grade, ≥ 98%). All low molecular mass (LMM) thiol compounds were also purchased from Sigma-Aldrich; their structures and abbreviations used throughout this manuscript are given in the Electronic Supplementary Material (ESM), Fig. S1. Formic acid (FA) was from Fluka, and methanol (MeOH) from Merck. Ultrapure Milli-Q water (> 18 MΩ.cm) was obtained through a Milli-Q Advantage A10 Ultrapure Water Purification System (Merck Millipore). Stock solutions of MeHg (4.0 mM) were prepared in 0.1 M HCl and preserved at + 4 °C, and the MeHg concentration was verified by combustion atomic absorption spectrometry measurements (Direct Mercury Analyzer DMA-80, Milestone). Thiol solutions (10 mM) were freshly prepared individually with deoxygenated Milli-Q water (purging 1 h with 300 mL min^−1^ N_2_ inside a glove box) under N_2_ atmosphere in a glove box. A 10-ml standard mixture consisting of 100 μM of each LMM thiol (13 thiols) was prepared by pipetting 100 μl of each LMM thiol solution (10 mM) and diluting with 8.7 ml deoxygenated Milli-Q water. In a 15-ml falcon tube (polypropylene), a solution containing 10 μM of each MeHg–thiol complex was prepared by addition of 1 ml of the mixed LMM thiols standard (100 μM of each thiol compound) with 8.3 ml deoxygenated Milli-Q water and 0.7 ml of MeHg stock solution (4.0 mM). The solution was rotated for 3 h prior to further dilution by 0.1% formic acid in deoxygenated Milli-Q water (pH = 2.7).

*Geobacter sulfurreducens* subsp*. sulfurreducens PCA* (American Type Culture Collection 51,573) was purchased from DSMZ. It was grown under N_2_ atmosphere at 28 °C and pH 6.8 in a defined growth medium as fully described by Schaefer et al. [20]. An experimental assay buffer typically used for Hg^II^ methylation experiment with *G. sulfurreducens* was adopted for this study [20, 21]. This buffer is composed of 2.1 MOPS, 0.03 MgSO_4_.7H_2_O, 0.1 KCl, 0.01 NaCl, NaH_2_PO_4_.H_2_O, 0.005 NH_4_Cl, 0.082 CH_3_COONa, 0.001 resazurin, all concentrations given in g/L, and 1 mM fumarate with pH adjusted to 6.8 with 10 M NaOH solution.

### Mass spectrometry and online preconcentration instrumentation

The mass spectrometry instrumentation setup consisted of a PAL HTC autosampler (CTC Analytics AG, Zwingen, Switzerland) with a cooled tray (+ 5 °C) connected to a Surveyor and an Accela LC-pump (Thermo FisherScientific, San Jose, CA, USA) dedicated to an online SPE cartridge and the analytical LC column, respectively, and a TSQ Quantum Ultra electrospray ionization triple quadrupole mass spectrometer instrument (Thermo Fisher Scientific, San Jose, CA, USA). Peek tubing (Ø 0.13 mm, Restek) was used to connect the different instrumental parts.

The general operating conditions for the instrument are given in Table 1. The fragmentation of each MeHg–thiol complex was studied by direct infusion to the TSQ Quantum Ultra instrument of solutions containing 10 μM of individual complexes using a flow rate of 25 μl min^−1^ combined with a 250 μl min^−1^ auxiliary flow rate of 50:50 MeHg: H_2_O with 0.1% FA. Full scan spectra of the complexes were recorded in both negative and positive ionization mode and parental masses of the complexes were identified based on Hg isotope pattern and the matching to theoretical masses of the complexes. Tube lens voltages and collision energy were optimized and thereafter set up individually for each complex in the selective reaction monitoring (SRM) method.

**Table 1:** Operating parameters for the instrument

Mass spectrometry	Thermo Scientific TSQ Quantum Ultra
Ion source	Heated electrospray ionization
Mode	Negative/positive
Sheath/auxiliary gas flow	60/25 (arbitrary units)
Collision gas	1.5 ml min^−1^ (argon)
Electrospray voltage	3.5 kV
Capillary/vaporizer temperature	325/225 °C
Scan range (*m*/*z*)	200–1000
HPLC column	Phenomenex, Kinetic Biphenyl (3.0 × 150 mm, 5 μm)
SPE cartridge	Water, Oasis WCX (2.1 × 20 mm, 15 μm)
Mobile phase	0.1% FA in water/MeOH (20–90%)
Injection volumes	10 μl or 1 ml

The sample loading procedure for online SPE preconcentration was previously described by Liem-Nguyen et al. [12]. Briefly, 1 mL of sample was loaded onto an online SPE cartridge with a switching-column array made up of a 6-port and a 10-port switching valve manufactured by Valco Instruments Co. The elution gradients used for the analytical columns with and without SPE are shown in Table S1 (see ESM). The recovery of MeHg–thiol complexes on different SPE materials was investigated based on their molecular structure which frequently contain alkyl, amino (NH_2_) and carboxylic (COOH) functional groups. The MeHg–thiol complexes have weak acid or base properties depending on pH of the solution. Thus, three different SPE cartridges of dimensions 2.1 × 20 mm, 15 μm were investigated at different pH: Hydrophilic-Lipophilic-Balanced (HLB) Oasis, Weak-Cation-Exchange (WCX) Oasis, and Weak-Anion-Exchange (WAX) Oasis, Water Scientific.

### Figures of merit determinations

The recovery of the online SPE procedure was calculated by comparing the analyte signal intensities of the complexes with SPE (0.1 μM and 1.0 ml injection) and without SPE (10 μM and 10 μl injection, i.e., direct injection onto the analytical column). Recovery was determined for both Milli-Q water with 0.1% FA and bacteria incubation media matrices. The LODs were calculated as 3 times the standard deviation (SD, *n* = 11) of the peak areas for Milli-Q water with 0.1% FA blank solutions divided by the sensitivity on a peak area per concentration basis (achieved from calibration curves). The peak area of each complex was the combined signal of the two product ions. The calibration curves were established with mixtures of the MeHg–thiol complexes with concentrations ranging from 1 to 50 nM of each complex in either MQ water or bacteria incubation medium. The method’s repeatability was evaluated with triplicate injections of standards. The complexes’ stability over time (30 days) at different storage temperature (room temperature (+ 22 °C), + 4 °C and − 20 °C) was investigated by preparing batches of mixed solutions containing 100 nM of each complex in 0.1% FA in 15-ml polypropylene falcon tubes. The samples with different storing temperature were all prepared on day 1 and sacrificed for analyses at regular time intervals during 30 days. The drift in instrument sensitivity during the 30-day investigation was corrected for by normalization of the peak areas of test samples to the corresponding peak areas of freshly prepared MeHg–thiol standards (100 nM each).

### Bacteria culture and incubation

Bacteria cells were harvested from the growth medium by centrifugation at mid-exponential phase under anaerobic condition. The harvested cells were then washed three times with 50 ml of assay buffer to ensure all adhering nutrients from the growth medium were washed off before the cells were re-suspended in assay buffer. The presence of MeHg–thiol complexes was investigated following the methylation of Hg^II^ in assays with 100 nM HgCl_2_ as described in Schaefer et al. [21] using a final assay of ~ 10^8^ cell ml^−1^, a bacterial population density typical of pure culture methylation experiments with *G. sulfurreducens* [20, 21, 36]. It is noteworthy that the methylation assay remained colorless throughout the 6- and 48-h incubation period at 30 °C, which indicated that the bacteria were not physiologically stressed and that the assays remained anaerobic. After 6 or 48 h of incubation, the assay medium was filtered through 0.2-μm syringe filters and the pH of the filtrate was adjusted to 2.7 by concentrated H_2_SO_4_ prior to analysis.

## Results and discussion

### Fragmentation of the MeHg–thiol complexes

The fragmentation of each MeHg–thiol complex was first investigated by directly introducing standard solutions to the mass spectrometer (without LC) in both negative and positive ESI mode with a mass scan ranging from 200 to 1000 *m*/*z*. The spectra of representative complexes are given in Fig. S2 (see ESM) and product ions obtained from each MeHg–thiol complex with the triple quadrupole mass spectrometer instrument is presented in Table 2. After the parental mass and product ions of each investigated complex were established, the selected reaction monitoring (SRM) mode was applied for quantification of the complexes. The SRM method was set to target the precursor ion and the two most abundant product ions for each complex using their respective optimal tube lens voltages and collision energies. The peak area signals corresponding to the transition of the parental ion to the two product ions of each complex were recorded and used for quantification of the MeHg−thiol complexes. For complex verification in the test solutions and real samples the peak area signal ratio of the two product ions was compared with the corresponding ratio of pure standard solutions in tune mode.

**Table 2:** Product ions obtained from each MeHg–thiol complex with the triple quadrupole mass spectrometer instrument (Thermo scientific TSQ Quantum Ultra). The product ions are sorted from high to low signal intensity (left to right) normalized to the signal of the fragment with highest intensity. Italic font indicates the two most frequently obtained product ions. The quantification method was set for the parental mass and the two most sensitive product ions of each complex; the third product ion was used for qualitative analysis

Complexes	Parental mass (*m*/*z*)	Tube lens (V)	Product ions (*m*/*z*) (relative intensity %, and optimum collision energy (V) are given in parenthesis)
MeHg-2MPA	320.9	− 92.4	*249.1 (90%, 20)*, 277.4 (5%, 10), 243.2 (2%, 79)
MeHg-SULF	356.9	− 75.6	*249.1 (100%, 23)*, 233.8 (18%, 41), 80.3 (13%, 37)
MeHg-SUC	364.9	− 67.3	*249.1 (95%, 23)*, 347.1 (23%, 14), 320.9 (10%, 5)
MeHg-Cyst	293.9	77.8	277.1 (100%, 6), *217.1 (33%, 24)*, 234.1 (14%, 6)
MeHg-Glyc	324.9	72.8	307.1 (90%, 5), *217.1 (33%, 35)*, 263.1 (30%, 11)
MeHg-Cys	337.9	98.6	321.1 (100%, 6), *217.1 (28%, 32)*, 234.1 (19%, 10)
MeHg-HCys	351.9	91.4	335.1 (100%, 10), 306.1 (90%, 12), 56.2 (70%. 18)
MeHg-Pen	365.9	81.8	349.1 (100%, 7), *217.1 (30%, 23)*, 305.1 (25%, 23)
MeHg-NACCys	379.9	84.3	362.1 (90%, 6), 321.0 (26%, 14), 56.2 (15%, 41)
MeHg-CysGly	394.9	90.0	378.1 (100%, 10), *217.1 (15%, 35)*, 321.0 (12%, 16)
MeHg-NACPen	407.9	90.0	390.2 (100%, 6), 70.4 (45%, 33), 349.1 (10%, 13)
MeHg-GluCys	466.9	113.1	321.0 (100%,16), *217.1 (19%, 39)*, 346.2 (15%, 17)
MeHg-GSH	524.0	109.1	378.1 (100%, 16), 395.2 (25%, 11), 345.1 (10%, 22)

**Fig. 1:**
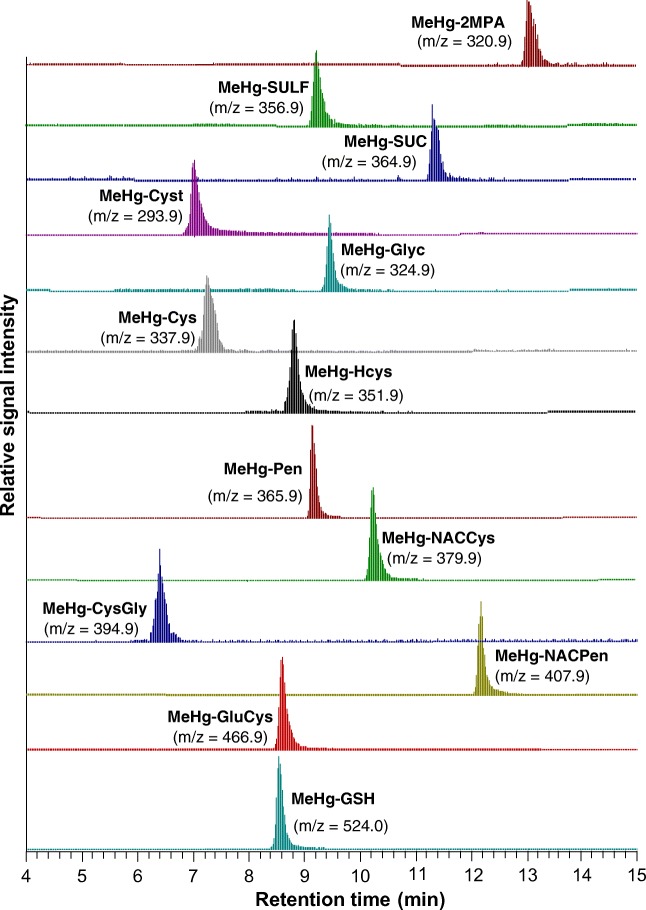
Typical LC-ESI-MS/MS chromatograms for parental ion measurements of the 13 MeHg–thiol complexes (100 nM each, pH = 2.7) using a Phenomenex Biphenyl Kinetic (3.0 × 150 mm, 5 μm) reversed-phase column fitted with a phenyl guard column (3.0 × 4.0 mm, 5 μm), after online preconcentration on a WCX SPE column with 1 mL injection volume

A typical LC-ESI-MS/MS chromatogram for the complexes is shown in Fig. 1. Among the 13 investigated MeHg–thiol complexes, 3 complexes, i.e., MeHg-2MPA, MeHg-SUC, and MeHg-SULF, showed higher signal intensity in negative ESI mode whereas the other 10 complexes gave higher signal intensity in positive ionization mode. The presence of protonated amino groups (-NH_3_^+^ or -NH_2_^+^-) generates positive charges and high signal intensity of the complex in positive ionization mode. The structures of 2MPA, SUC and SULF do not contain amino groups but carboxylic groups (-COOH) which provide a negative charge when deprotonated and thereby a higher intensity in negative mode (ESM Fig. S1). The parental mass of the complexes was confirmed based on the matching of the Hg isotope pattern and matching between their theoretical and measured molecular mass (*m*/*z*) corresponding to the ionization mode. The two fragments *m*/*z* 217 (CH_3_–Hg^+^) and 249 (CH_3_Hg–S^+^) were the most commonly observed and were obtained for MeHg-Cyst, MeHg-Glyc, MeHg-Cys, MeHg-Pen, MeHg-CysGly and MeHg-GluCys, MeHg-2MPA, MeHg-SULF, and MeHg-SUC. These 9 (of the 13) complexes thus produced common product ions specific for MeHg–thiol complexes with preserved CH_3_–Hg or CH_3_Hg–S bonds. A similar fragmentation transition was previously observed during the determination of LMM thiols by ESI-MS/MS using p-(Hydroxymercuri) Benzoate, a probe containing Hg, as a derivatization reagent [12].

### Online SPE preconcentration and matrix effect

**Fig. 2:**
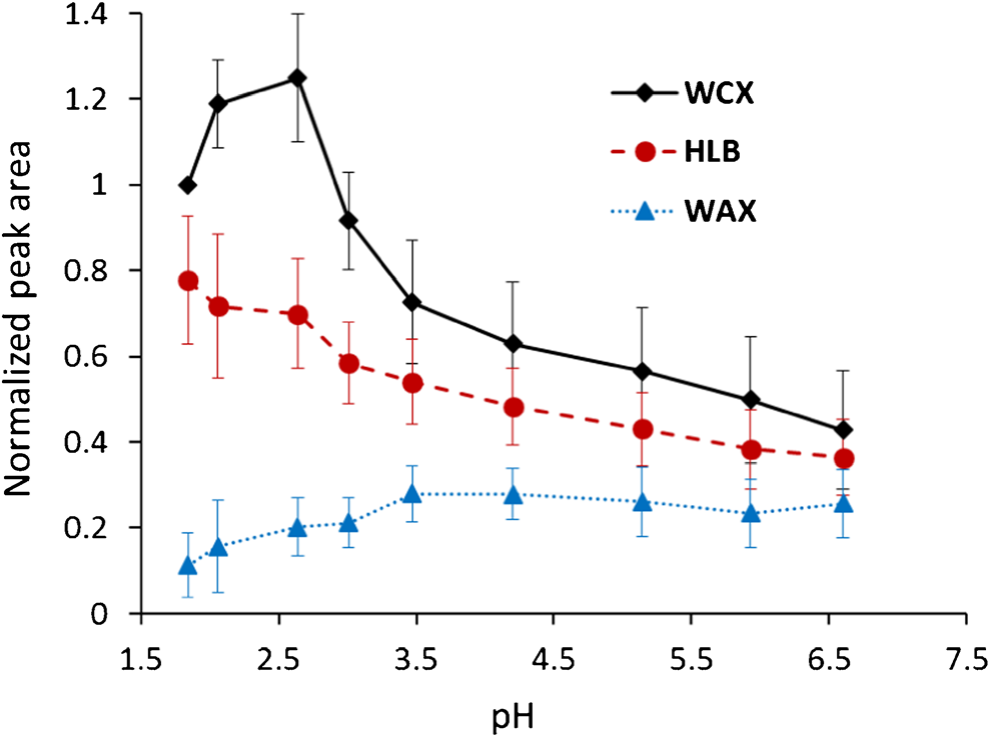
Average normalized peak area of MeHg–thiol complexes (100 nM each) by SPE preconcentration using three different SPE phases (WCX, HLB, WAX). The peak areas are normalized to the ones obtained at pH of 1.84 with the WCX cartridge for each complex. The samples were prepared in 0.1% FA Milli-Q water and pH was adjusted by varying additions of 1 M of NaOH or H_2_SO_4_. The error bars represent confidence intervals (*p* = 0.05, *n* = 26) for the 13 investigated complexes

Three different Waters Oasis SPE cartridges (WCX, WAX, HLB) with the same structure basis were investigated [37]. Among those SPE cartridges, the HLB is the “base material” which offers both hydrophilic and lipophilic interactions through 2-pyrrolidone, and phenyl and alkyl functional groups. The WCX and WAX cartridges are modified versions of the HLB with addition of extra carboxylic and diamino functional groups, respectively. The results showed that, in general, the WCX cartridge offered highest retention efficiency, on average 1.5-fold and 4-fold higher than the HLB and WAX, respectively, at the corresponding optimal pH for each SPE cartridge. The optimal pH range for the WCX cartridge was between 2.2 and 2.8 (Fig. 2). This could be explained by the fact that at pH 2.2 to 2.8, the carboxylic group of the WCX SPE and the amino group of MeHg–thiol complexes will be present as protonated forms, i.e., –COOH (pKa ~ 5) and –NH_3_^+^ (pKa = 8 to 11), respectively. The hydrogen bonding and polar interaction between the –COOH and –NH_3_^+^ functional groups enhance the retention of the complexes on the WCX cartridge. For the WAX cartridge, at a low pH (pH < 3) repulsive forces appear between the protonated positively charged diamino group of WAX (pKa~6) and the amino group of the complexes. The recovery of the complexes on the WAX cartridge increased with pH which can be explained by a deprotonation of the –COOH (pKa = 2 to 5) groups of the MeHg–thiol complexes. Our initial results from offline SPE experiments demonstrated a strong retention of the investigated complexes (MeHg-Cys and MeHg-Pen) on a strong cation exchange cartridge (MCX, Water Oasis). However, a very strong elute solution (100 mM ammonium acetate in acetone) was needed to elute the complexes out of the cartridge. The MCX cartridge is therefore difficult to apply with the online SPE and ESI-MS/MS system, because of the high salt concentration and organic solvent strength constrains, and the investigation with online MCX cartridge was thus terminated.

### MeHg–thiol complexes synthesis and preservation

**Fig. 3:**
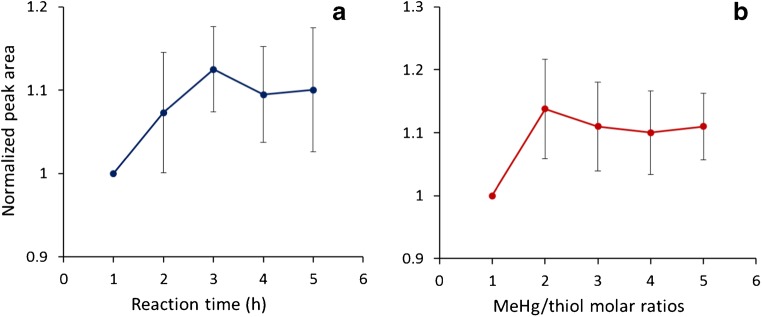
The average normalized peak area of 13 investigated MeHg–thiol complexes in the optimization of complexes synthesis following reaction time (**a**) and MeHg to thiol molar ratios (**b**). The peak areas are normalized to the ones obtained at the reaction time of 1 h (**a**) and the MeHg to thiol molar ratio of 1 (**b**). The error bars represent confidence interval (*p* = 0.05, *n* = 26) for 13 investigated complexes

The MeHg–thiol complexes were synthesized with an excess stoichiometry of MeHg (normally using a MeHg to thiol molar ratio of 2). The advantage of this procedure is that the mixed MeHg–thiol complexes standard (13 thiols) can be accurately prepared because MeHg is always available for complete reaction with all thiol ligands. The time needed for completing the reaction between MeHg and thiols was investigated. Figure 3a shows that when MeHg was mixed with thiol ligands at a molar ratio of 2:1 ([MeHg]/[thiol] = 2), the reaction for formation of the corresponding MeHgSR(aq) complexes was close to steady-state after 1 h. From 1 to 3 h, the average signal of the complexes increased on average by approximately 12%, but not statistically significant, and was followed by steady-state conditions at extended reaction times. Fast reactions of MeHg and Hg^II^ with LMM thiols have been reported in previous studies, with the average life time of ~ 30 s and 0.01 s, respectively [24–26]. The MeHg to thiol molar ratio was varied from 1 to 5 (3-h reaction time), Fig. 3b, and the results indicated that with a molar ratio of 2 or higher the MeHg–thiol complexes’ signal plateaued. Thus, a reaction time of 3 h and a MeHg to thiol molar ratio of 2 was applied for further synthesis of the MeHg–thiol complexes.

It is well known that reduced thiol groups are highly reactive and unstable, and the stability of MeHg–thiol complexes is not well-characterized. We analyzed series of sample batches stored in dark at different temperature (ambient (i.e., + 22 °C), + 4 °C and − 20 °C) in regular 15-ml polypropylene falcon tubes. The results showed that all investigated complexes were stable for at least 2 weeks at all conditions, and MeHg-2MPA, MeHg-SUC, MeHg-SULF, MeHg-NACPen, and MeHg-GSH were stable for at least 30 days. Different temperatures did not significantly affect the complexes’ stability as can be observed in Fig. S3 (see ESM). A previous study has shown that LMM thiols derivatized to O_2_C_7_H_5_Hg–thiol complexes are stable for up to a month in most cases at + 4 °C and − 20 °C [12]. This indicates that metal ions (in this case MeHg) efficiently stabilize the RSH group of LMM thiol compounds, reducing its reactivity for oxidation/degradation.

### Figures of merit

Without using SPE preconcentration, the LODs for the MeHg–thiol complexes ranged from 0.5 to 32 nM, which is comparable to previous methods using LC-ICPMS [35]. In combination with online SPE preconcentration, the method achieved the lowest LODs for the determination of MeHg–thiol complexes up to date with a range from 0.012 to 0.53 nM and average of 0.16 nM (Table 3). By using SPE preconcentration, the LODs were improved by on average a factor of 70 as compared to without SPE. This improvement was lower than the theoretical preconcentration factor (i.e., 100), which is explained by a non-quantitative recovery of the complexes (average 86% recovery) and an increased signal to noise level. Overall, the LODs were lower for the complexes measured in positive ESI mode (average 0.12 nM) compared to the ones measured in negative ESI mode (average 0.34 nM) with the exception of MeHg-Glyc (0.35 nM) and MeHg-HCys (0.42 nM) which had comparably high LOD despite being analyzed in positive mode (Table 3). The complexes analyzed in positive ESI showed about 15-fold higher signal intensity compared to the ones analyzed in negative ESI; however, the noise level in the positive mode was about 4-fold higher.

**Table 3:** Limits of detection (LODs) and analytical reproducibility (given as the relative standard deviation, RSD) achieved for each complex with optimized conditions without and with preconcentration by solid phase extraction (SPE)

Complexes	Ionization mode	Without SPE		With SPE	
		LODs^a^ (nM)	RSD^b^ (%)	LODs (nM)	RSD (%)
MeHg-GSH	+	0.50	2.8	0.012	3.3
MeHg-Pen	+	0.75	5.2	0.015	11
MeHg-CysGly	+	1.8	3.9	0.035	9.8
MeHg-NACCys	+	3.4	4.5	0.038	6.4
MeHg-NACPen	+	2.8	8.4	0.055	9.5
MeHg-GluCys	+	2.5	7.3	0.065	10
MeHg-Cys	+	6.0	5.7	0.087	12
MeHg-SULF	–	17	8.7	0.14	9.6
MeHg-Cyst	+	15	5.5	0.15	7.5
MeHg-2MPA	–	20	6.7	0.25	8.3
MeHg-Glyc	+	21	7.6	0.35	8.3
MeHg-HCys	+	25	8.9	0.42	9.6
MeHg-SUC	–	32	8.6	0.53	11
Average		11.4	6.4	0.16	8.9

Milli-Q water (0.1%FA) without SPE and with SPE preconcentration, respectively

^a^The LODs were determined as 3σ of 11 blank replicates

^b^The RSD were established with 5000 and 100 nM standard solutions in

The recoveries for the MeHg–thiol complexes when using the SPE preconcentration procedure compared to direct injection to the LC column was on average 86 ± 8% (range 75–102%) for a Milli-Q water matrix (0.1% FA) and 78 ± 8% (range 71–85%) for the bacteria incubation assay medium (0.1% FA) (ESM Table S2). Therefore, about 10% and 20% of the complexes was lost during the preconcentration for Milli- Q water matrix and the bacteria incubation medium, respectively. The matric effects from the bacteria incubation assay medium on the quantification of the complexes were investigated by comparing signal to noise ratio (S/N) between Milli-Q water (pH = 2.7) and the incubation medium (pH = 2.7) matrices containing 20 nM of each MeHg–thiol complex. The results showed that the difference in S/N between the two matrices was less than 30% for MeHg-SUC, MeHg-Glyc, MeHg-Pen while the S/N was reduced 2 to 8 folds for the other complexes. There were no significant differences in the signal intensities of the complexes in the two matrices; therefore, the reduced S/N ratios were caused by an increase in the background level with the incubation medium. To minimize the effect of the medium composition and recovery of the LC column on the quantification of the complexes, calibration curves were established using the incubation media as matrix for the standard solutions in this case.

### Determination of MeHg–thiol complexes in *G. sulfurreducens* assay

Adediran et al. recently reported biosynthesis and excretion of various LMM thiols in *G. sulfurreducens* bacteria cultures reaching up to about 120 nM total thiol concentrations in the extracellular medium [11]. To demonstrate the potential of the developed method for the determination of MeHg–thiol complexes in the presence of bio-synthesized LMM thiols, 400 nM of MeHg was added to filtered extracellular assay medium collected after incubation for 6 h with live *G. sulfurreducens* cells. The samples were equilibrated in an N_2_ filled glove box for 3 h and the pH was then adjusted to 2.7 by sulfuric acid prior to analysis. Seven different MeHg–thiol complexes were detected in the samples, MeHg-Cyst, MeHg-CysGly, MeHg-Cys, MeHg-GluCys, MeHg-NACCys, MeHg-GSH, and MeHg-Pen with total concentration of 44 nM (ESM Table S3). Thus, only about 10% of added MeHg was detected as complexes with the LMM thiols, which could be explained by a lower concentration of biosynthesis LMM thiols compared to the added MeHg and/or the reaction of MeHg with other thiols or functional groups in solution.

The method was then applied to identify and quantify extracellular MeHg–thiol complexes at 6 and 48 h in active *G. sulfurreducens* incubation assays amended with 100 nM of HgCl_2_. The results in Table 4 show that, with the 6-h incubation, 4 complexes, MeHg-Cyst, MeHg-Cys, MeHg-Pen, and MeHg-CysGly, were detected at concentrations ranging from 1.3 to 6.1 nM. For the 48-h incubation, 6 MeHg-LMM thiols were detected including the 4 mentioned compounds and MeHg-GluCys and MeHg-GSH with concentrations ranging from 0.5 to 5.6 nM.

**Table 4:** Measured concentrations (nM) of extracellular MeHg–thiol complexes and LMM thiol compounds in assay medium from *G. sulfurreducens* incubations with amendment of 100 nM of Hg. The cell density was ~ 10^8^ cell ml^−1^ and constant during the incubation time

	6-h incubation		48-h incubation	
Complex	MeHg–thiol complex (nM)	Corresponding LMM thiol compound (nM)	MeHg–thiol complex (nM)	Corresponding LMM thiol compound (nM)
MeHg-Cys	6.1 ± 0.5	30 ± 6	5.1 ± 0.7	33 ± 5
MeHg-Cyst	5.2 ± 0.7	12 ± 2	5.6 ± 0.4	14 ± 2
MeHg-Pen	3.2 ± 0.3	14 ± 3	3.0 ± 0.5	16 ± 3
MeHg-CysGly	1.3 ± 0.2	5.3 ± 1.5	1.3 ± 0.2	7.3 ± 1.5
MeHg-GluCys	–	2.6 ± 1.3	0.5 ± 0.07	6.2 ± 1.6
MeHg-NACCys	–	1.8 ± 0.6	–	2.6 ± 0.4
MeHg-Hcys	–	1.2 ± 0.4		1.5 ± 0.3
Total	15.8	67	16	81

The stability constants of MeHg to LMM thiols are not well established and to gain further insight in the binding strength of different LMM thiols to MeHg, separate determinations of the LMM thiol compounds that were synthesized and exported by the bacteria into the extracellular solution were also done using the methodology reported by Liem-Nguyen et al. [12]. Seven different LMM thiols were detected, Cys, Cyst, Pen, CysGly, GluCys, NACCys, and HCys, with a total concentration of 67 nM and 81 nM for 6- and 48-h incubation, respectively (Table 4). A similar range of extracellular LMM-thiol concentration was also reported by Adediran et al. for the same type of bacterium and similar bacteria density of ~ 10^8^ cell ml^−1^ [11]. There was no significant difference (*t* test, *p* > 0.05) between the concentrations of MeHg-Cys and MeHg-Cyst complexes even though the total Cys concentration was about 2.5 times higher than the total Cyst concentration in the assay systems. This could be explained by a competition between Hg^2+^ and MeHg for LMM thiol ligands. Hg^2+^ has a stronger binding affinity to LMM thiols than MeHg, and thus influences the formation of MeHg–thiol complexes [23, 24]. The stability constants for the formation of Hg(SR)_2_ complexes increase in the order Hg(GluCys)_2_ > Hg(Pen)_2_ > Hg(Cys)_2_ > Hg(CysGly)_2_ > Hg(Cyst)_2_ [23]. The lower constant for Hg(Cyst)_2_ compared to Hg(Cys)_2_ can thus lead to a higher concentration of Cyst, compared to Cys, available for formation of complexes with MeHg when both thiols are present in the same concentration. In the system where only MeHg was added the concentration of MeHg-Cys was 1.6 times higher than MeHg-Cyst (ESM Table S3) which further supports the explanation of competition from Hg^II^. The discrepancy in molar ratios of Cys/Cyst = 2.5 and MeHg-Cys/MeHg-Cyst = 1.6 in the system without Hg^II^ further implies a stronger binding affinity of MeHg to Cyst compared to Cys.

Even though NACCys and Hcys were detected their corresponding complexes with MeHg were not detected. This result could be explained by relatively low concentrations of these LMM thiols compared to the other ones yielding lower than LOD concentrations for the corresponding MeHg complexes. Overall, the results illustrate how the developed method is useful to investigate the stability of MeHg–thiol complexes and direct measurements of MeHg–thiol complexes (and the corresponding LMM thiol) is important for accurate determinations of the thermodynamic stability constant of MeHg–thiol complexes.

## Conclusion

The developed method achieved excellent detection limits for a relatively large number of MeHg–thiol complexes ranging from 12 to 530 pM. The method was carefully optimized and successfully applied to quantify various MeHg–thiol complexes produced in bacteria incubation assays. With an amendment of 100 nM of Hg^II^, relevant to contaminated environment conditions, 5 different MeHg–thiol complexes were quantified with concentrations ranging from 0.5 to 6.1 nM. This is the first time MeHg–thiol complexes are directly quantified in methylating bacteria cultures. The method offers a unique opportunity to improve our understanding of MeHg biogeochemistry, especially bioaccumulation and bio-toxicity processes in aquatic organisms and human organs.

## Electronic supplementary material

ESM 1 (PDF 484 kb)
