# Supplementary material for: Determination of picomolar levels of methylmercury complexes with low molecular mass thiols by liquid chromatography tandem mass spectrometry and online preconcentration
Source: Anal Bioanal Chem. 2020 Jan 16;412(7):1619–28. doi: 10.1007/s00216-020-02389-y (PMC7026298; doi:10.1007/s00216-020-02389-y)
Supplement: Supplementary file 1 — (PDF 484 kb) [file 216_2020_2389_MOESM1_ESM.pdf]

Analytical and Bioanalytical Chemistry

Electronic Supplementary Material

Determination of picomolar levels of methylmercury complexes with low molecular mass thiols by liquid chromatography tandem mass spectrometry and online preconcentration

Van Liem-Nguyen, Hoang-Tung Nguyen-Ngoc, Gbotemi A. Adediran, Erik Björn

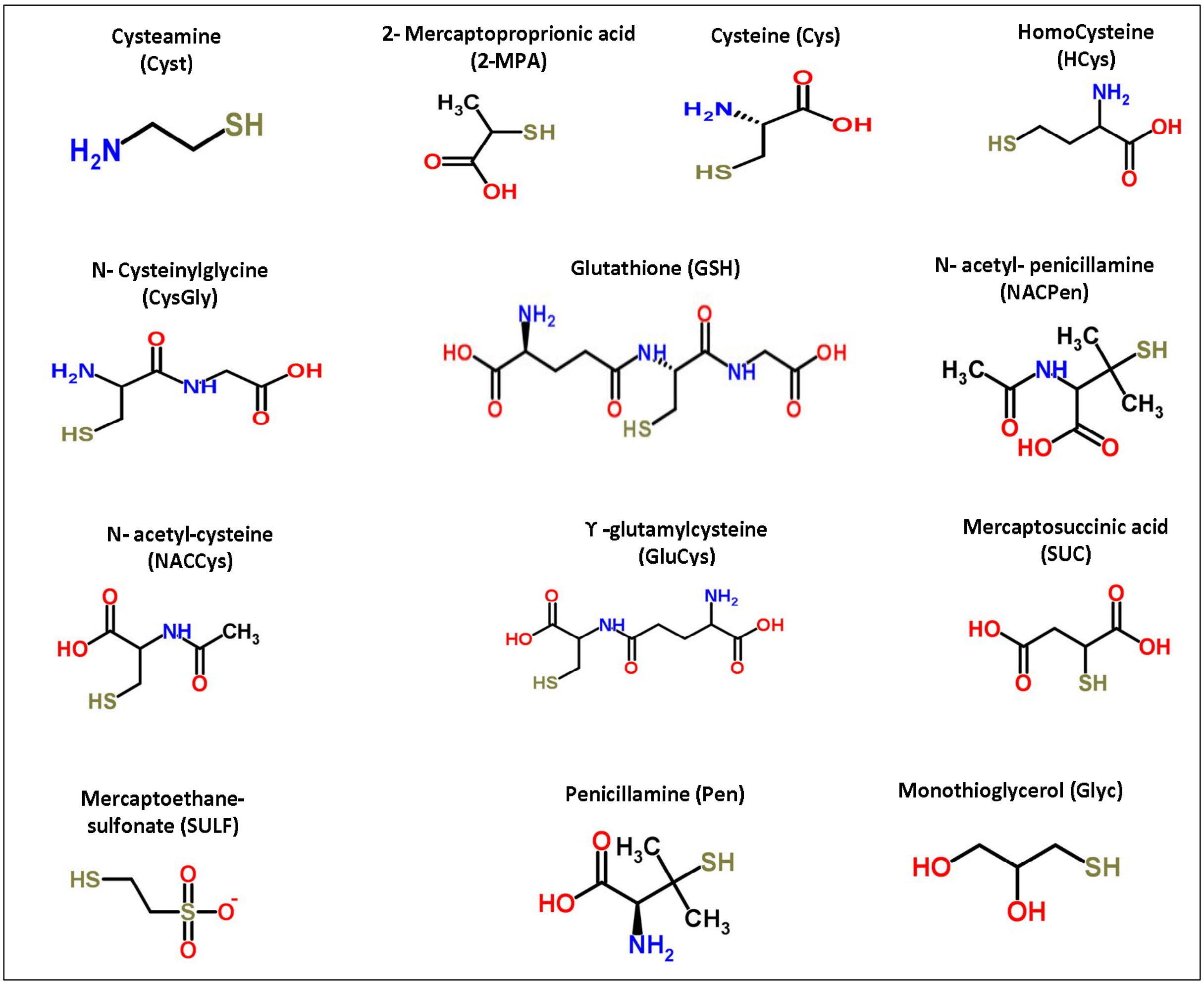

Fig. S1 Molecular structure and abbreviation name of investigated LMM thiols. In complexation with MeHg, MeHg replaces the H of the thiol group. Adapted and revised from Liem-Nguyen et al. [1]

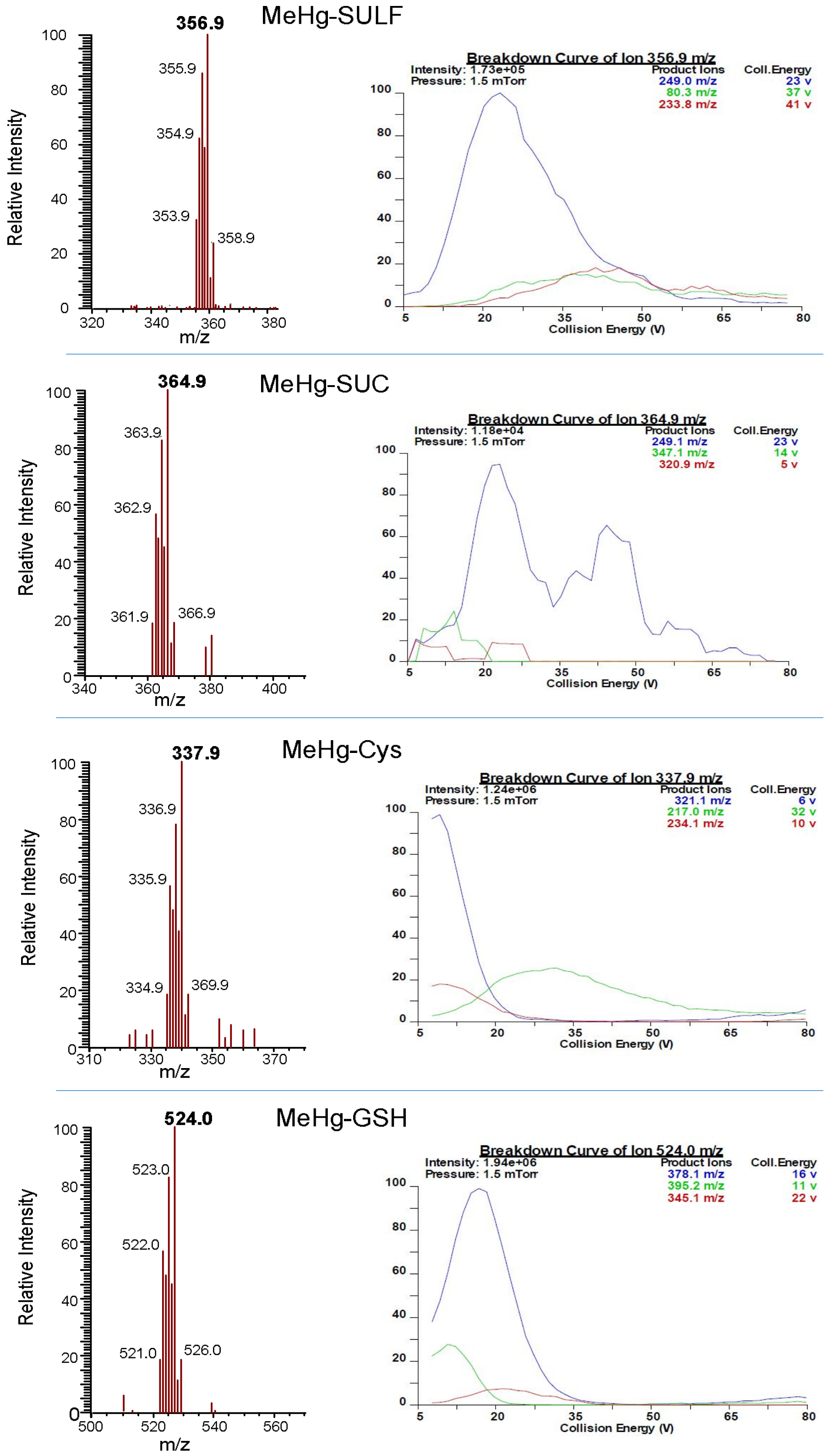

Fig. S2 Showing mass spectrometry spectra and fragmentation of representative MeHg–thiol complexes in negative (MeHg-SULF and MeHg-SUC) and positive (MeHg-Cys and MeHg-GSH) ESI modes

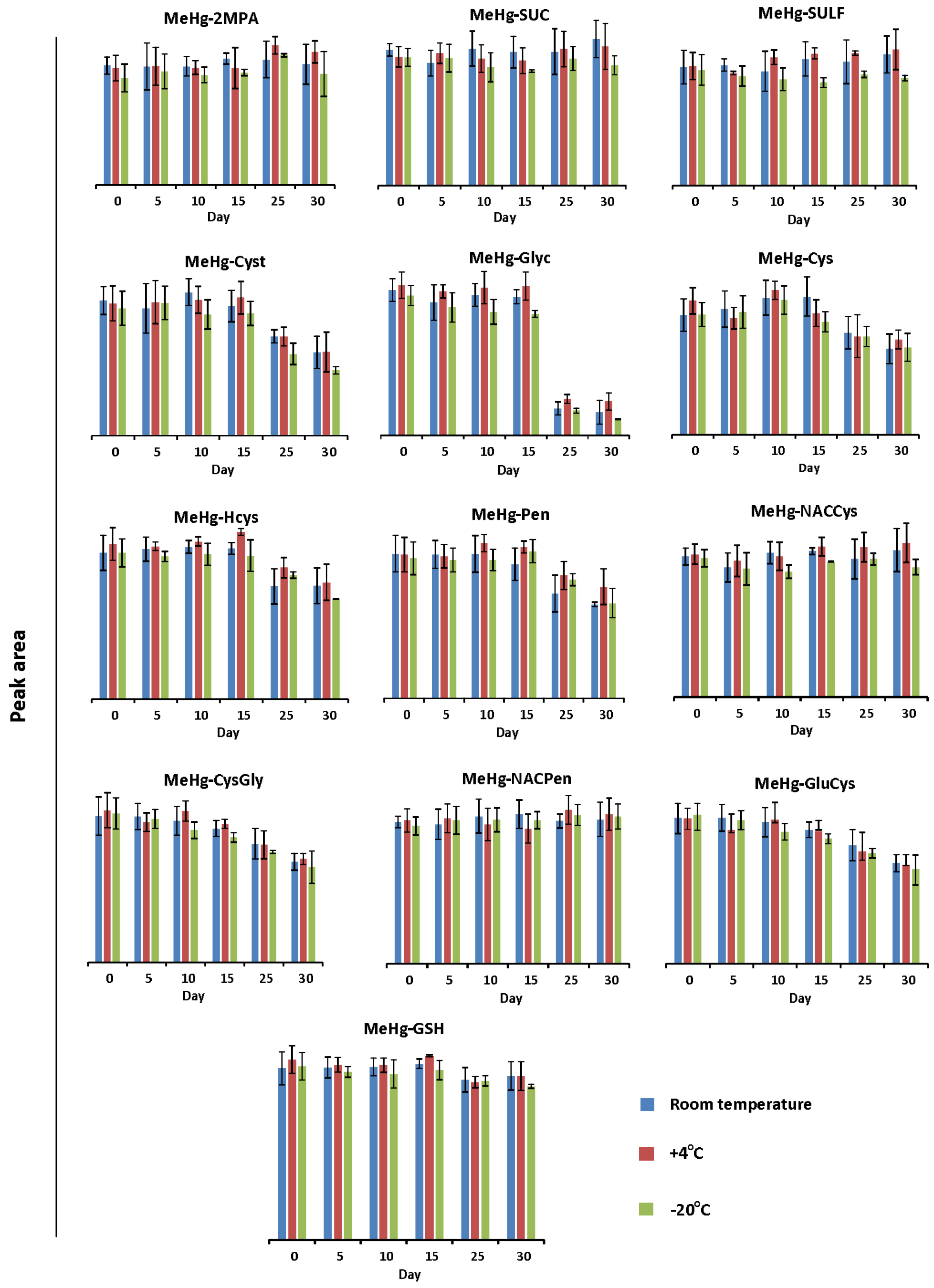

Fig. S3 Stability over time of MeHg–thiol complexes (100 nM of each) in Milli-Q water with 0.1% formic acid (pH=2.7) in polypropylene containers at different temperatures. Samples were analyzed using the SPE online preconcentration procedure. The instrumental drift was corrected by running a set of freshly prepared MeHg–thiol complexes

Table S1 The gradient of the analytical LC column (Acela Pump) for without SPE and with SPE 1 ml injection loops

Time (min)	Without SPE			Time (min)	SPE 1 ml		
	0.1%FA H_2O	0.1%FA MeOH	Flow rate µl min^{-1}		0.1%FA H_2O	0.1%FA MeOH	Flow rate µl min^{-1}
0	90	10	300	0	90	10	300
1	90	10	300	2	90	10	300
12	10	90	300	2.1	90	10	300
14	10	90	300	12	10	90	300
16	90	10	300	14	10	90	300
20	90	10	300	14.1	90	10	300
				20	90	10	300

Table S2 The SPE recoveries of each MeHg–thiol complex under various matrix conditions

Complexes	SPE recovery for Milli-Q water matrix* (%) (±SD)	SPE recovery for bacteria incubation media matrix* (%) (±SD)
**MeHg-2MPA**	102 ± 10	89 ± 7
**MeHg-SULF**	77 ± 8	81 ± 12
**MeHg-SUC**	90 ± 7	95 ± 14
**MeHg-Cyst**	87 ± 5	73 ± 6
**MeHg-Glyc**	76 ± 9	72 ± 13
**MeHg-Cys**	85 ± 10	76 ± 11
**MeHg-Hcys**	89 ± 7	71 ± 9
**MeHg-Pen**	92 ± 8	75 ± 8
**MeHg-NACCys**	85 ± 6	76 ± 5
**MeHg-CysGly**	75 ± 9	71 ± 10
**MeHg-NACpen**	93 ± 11	81 ± 9
**MeHg-GluCys**	84 ± 9	72 ± 8
**MeHg-GSH**	90 ± 4	83 ± 9
**Average**	**86**	**78**
**SD**	**8**	**7**

*The recovery of each MeHg–thiol complex in Milli-Q water or incubation media using SPE preconcentration (V_{inj} = 1 mL at 50 nM each) compared to directly injected to analytical column (V_{inj} = 10 µL at 5 µM each). For sample with SPE, pH of sample was adjusted to optimal pH of 2.7 for WCX SPE cartridge while without SPE pH of sample was not measured.

Table S3 Measured concentrations of MeHg–thiol complexes in the extracellular medium collected from G. sulfurreducens assays (~ 10^8 cell ml^{-1}, 6 hrs incubation) amended with 400 nM MeHg

**Complex**	Concentration (nM)
**MeHg-Cys**	15.5 ± 3.3
**MeHg-Cyst**	9.8 ± 2.5
**MeHg-CysGly**	7.7 ± 1.3
**MeHg-GluCys**	5.2 ± 1.3
**MeHg-NACCys**	3.2 ± 1.2
**MeHg-Pen**	2.6 ± 0.8
**Total**	**44**

References

[1] Liem-Nguyen V, Bouchet S, Björn E. Determination of Sub-Nanomolar Levels of Low Molecular Mass Thiols in Natural Waters by Liquid Chromatography Tandem Mass Spectrometry after Derivatization with p-(Hydroxymercuri) Benzoate and Online Preconcentration, Anal Chem. 2014; 87(2): 1089-1096.
